# Optimization of differentiation protocols of dental tissues stem cells to pancreatic β-cells

**DOI:** 10.1186/s12860-022-00441-6

**Published:** 2022-09-20

**Authors:** Riham M. Aly, Hadeer A. Aglan, Ghada Nour Eldeen, Hanaa H. Ahmed

**Affiliations:** 1 Stem Cells Laboratory, Center of Excellence for Advanced Sciences, National Research Centre, 33 El Buhouth St., Dokki, Giza, 12622 Egypt; 2 Basic Dental Science Department, Oral Medicine & Dentistry Research Institute, National Research Centre, 33 El Buhouth St., Dokki, Giza, 12622 Egypt; 3 Hormones Department, Medicine Research and Clinical Studies Research Institute, National Research Centre, Dokki, Giza, Egypt; 4 Molecular Genetics & Enzymology Department, Human Genetic & Genome Research Institute, National Research Centre, Dokki, Giza, Egypt

**Keywords:** Dental stem cells, Pancreatic beta cells, Differentiation, Insulin producing cells

## Abstract

**Background:**

Despite the recent progress in the differentiation strategies of stem cells into pancreatic beta cell lineage, current protocols are not optimized for different cell types. The purpose of this study is to investigate and compare the ability of stem cells derived from dental pulp (DPSCs) and periodontal ligament (PDLSCs) as two anatomically different dental tissues to differentiate into pancreatic beta cells while assessing the most suitable protocol for each cell type.

**Methods:**

DPSCs & PDLSCs were isolated and characterized morphologically and phenotypically and then differentiated into pancreatic beta cells using two protocols. Differentiated cells were assessed by qRT-PCR for the expression of pancreatic related markers Foxa*-2, Sox-17, PDX-1, Ngn-3, INS and Gcg.* Functional assessment of differentiation was performed by quantification of Insulin release via ELISA.

**Results:**

Protocol 2 implementing Geltrex significantly enhanced the expression levels of all tested genes both in DPSCs & PDLSCs. Both DPSCs & PDLSCs illustrated improved response to increased glucose concentration in comparison to undifferentiated cells. Moreover, DPSCs demonstrated an advanced potency towards pancreatic lineage differentiation over PDLSCs under both protocols.

**Conclusion:**

In conclusion, the current study reports the promising potential of dental derived stem cells in differentiating into pancreatic lineage through selection of the right protocol.

## Introduction

Although, Diabetes Mellitus (DM) is considered one of the most prevalent disease, yet to date there is no definitive cure [1]. In fact current modalities to treat DM are mainly short termed and rely mostly on insulin injections to control blood glucose levels especially for Type I DM. Such strategies, however are inefficient since sufficient insulin secretion is not always achieved which could lead to hyper-glycaemia with successive complications. Other long term modalities include islet transplantation which present with significant limitations such as limited cadaveric donors and possible immune rejection in addition to subjecting the patients to prolonged periods of immunosuppression [2].

Recently, stem cell based therapies have emerged as a promising treatment option especially for diseases like DM which exclusively involve single cell; β-cell. Stem cells are renowned for their regenerative abilities and immunomodulatory characteristics. They are defined as undifferentiated cells that possess the capability of self-renewal, unlimited proliferation and the ability to differentiate into a large variety of cells depending on their origin and potency [3]. They primarily act through differentiation into Insulin Producing Cells (IPCs), immunomodulation and by eliciting a paracrine effect [4].

In fact over the past few years, stem cells from different sources including embryonic stem cells (ESCs), mesenchymal stem cells (MSCs) and induced pluripotent stem cells (iPSCs) have been reported to successfully differentiate into IPCs. However, MSCs are generally considered the cells of choice in the field of stem cell based therapies and regenerative medicine since they can be isolated from various sources in addition to their feasibility of culture in vitro, plasticity, and outstanding immunological properties [5].

Among the different sources of MSCs, are bone marrow, adipose tissue, umbilical cord and dental tissues. Dental tissues are one of the most readily available and accessible sources of MSCs, that represent minimal risk to the donor and present no ethical concerns. Extracted molars or exfoliated deciduous teeth are usually discarded during routine dental visits. Dental derived stem cells have neural crest origins and can thus be differentiated into various cell types. Moreover, they are suitable candidates for autologous stem cell transplantation. Such properties distinguish them from other mesenchymal stem cells [6–8].

Although, several protocols for derivation of IPCs from various MSCs sources have been previously described, however only a few have reported the use of dental derived stem cells [9–13] Govindasamy et al., [11] was the first to report the ability of dental pulp stem cells (DPSCs) of deciduous teeth to trans-differentiate into pancreatic cell lineage and proposed that dental stem cells as a rather non-controversial source of stem cells which could be safely employed in autologous stem cell based therapies targeting diabetes. Shivakumar et al., [9] also recently assessed the differentiation potential of stem cells isolated from three sources of dental tissues into pancreatic beta cells. Their results were promising and helped to identify dental derived stem cells from a single donor as a potent source for of beta cells. It has also been suggested that DPSCs represent a leading candidate for beta cell generation since they share similarities to neural cells together with pancreatic beta cells [14].

Differentiation protocols are generally designed to mimic normal human pancreatic development through manipulating signaling pathways via the administration of various growth factors and small molecules over a period of time ranging from a few days to several weeks [15]. Nevertheless, despite the presence of various protocols, optimization of the differentiation protocols according to the cell type is lacking. Such optimization should be carefully investigated in order to translate these protocols clinically. Moreover, full understanding of different cell types, differentiation protocols and mechanisms of differentiation should be thoroughly studied.

In this study we applied two different and well established protocols for generation of pancreatic β cells originally designed for other MSCs types, on stem cells isolated from two different dental tissues. We hypothesized that differentiation protocols need to be carefully selected and optimized according the cells subjected to differentiation. To investigate this hypothesis, DPSCs & PDLSCs were subjected to the first protocol of culture conditions comprising exendin-4 along with increasing concentrations of nicotinamide and β-mercaptoethanol [16]. In the second protocol [17], the effect of Geltrex as an extracellular matrix on the generation of IPCs from DPSCs & PDLSCs was evaluated together with other β cell induction factors. The purpose of this study is therefore designed to investigate and compare the ability of stem cells derived from dental pulp (DPSCs) and periodontal ligament (PDLSCs) as two anatomically different dental tissues to differentiate into pancreatic beta cells while assessing the most suitable protocol for each cell type.

## Materials and methods

### Isolation and preparation of human dental pulp stem cells (DPSCs)

Human third molar teeth indicated for extraction were collected from patients aged (16-24 years) after obtaining an informed consent from them. Extraction of teeth was carried out at the National Research Centre’s clinics under the approval of the Centre’s ethical committee (approval no. 19109). All methods were performed in accordance with the guidelines and regulations of the Declaration of Helsinki. The extracted teeth were kept in Dulbecco modified Eagle’s media (Lonza) to which penicillin/streptomycin (Lonza) and 10% FBS (Life Science Group) were added. The teeth were split open and the pulp was gently removed using sterile dental probe. Collected pulp tissue was washed in PBS (Lonza) two times to ensure removal of any exogenous debris. Tissues were then carefully dissected into small pieces using sterile scissors. 0.2% collagenase II (Serva Electrophores, Germany) solution was then added to the dissected pulp tissue and shaken in a water bath shaker at 37 °C for 30 minutes. The isolated dental pulp cells were cultured in fresh DMEM media supplemented with 10% FBS and incubated at 37 °C and 5% CO_2_. Cells were selected on the basis of their ability to adhere to the tissue culture plastic; non adherent hematopoietic cells were removed during medium replacement after approximately 5 days in culture. Medium was changed twice per week thereafter. Dental pulp cells were subsequently detached using 0.25% trypsin/1 mM ethylene diaminetetraacetic acid (EDTA) re-plated at 5 × 10^3^ cells/cm^2^ and cultured then passaged until reaching 70% confluency.

### Isolation of human periodontal ligament stem cells (PDLSCs)

Healthy human impacted third molars (free of inflammation) were placed in 50 ml sterile polypropylene tube containing alpha modified Eagle’s medium (alpha-MEM) supplemented with antibiotics (100 U/ml penicillin and 100 mg/ml streptomycin) immediately after extraction. Periodontal ligament tissue was removed from the surface of the root by sterile lancet, rinsed five times by PBS supplemented with penicillin and streptomycin. Periodontal ligament pieces were mixed together and minced into tiny pieces, then digested in a solution of 2 mg/ml collagenase type I (Serva Electrophores, Germany) for 30 min at 37 °C in a water bath. Single cell suspensions were seeded into a T-25 Flask with alpha-MEM (Lonza), supplemented with 15% fetal calf serum (Lonza), 400 mmol/ml L-glutamine, 20,000 U/ml penicillin and 20,000 ng/ml streptomycin and then incubated in a humidified atmosphere of 5% CO_2_ at 37 °C. The flasks were periodically checked every 24 hours by the phase contrast inverted microscopy and the culture medium was changed 3-times per week, the cells were sub-cultured when reached about 70% confluency by using 0.25% trypsin and 0.02% EDTA.

### Flow cytometry analysis

To ensure that the cells in cultures are MSCs and maintain their phenotype after propagation in culture [18], they were characterized by flow cytometry analysis for MSCs specific markers (CD14 and CD90). The PE conjugated-CD 14 and CD 90 antibodies were procured from Beckman Coulter Co. (USA) and R&D Systems (UK) respectively. The cells were incubated with the antibody against each of the surface markers for 20 min at 25 °C followed by flow cytometry analysis using Beckman Coulter Elite XL, USA instrument.

### Differentiation of DPSCs and PDLSCs into pancreatic β cell lineage

Cells of the third passage from both DPSCs and PDLSCs groups were induced to differentiate into pancreatic β cell lineage using two different protocols.

#### Protocol 1

**Fig. 1:**
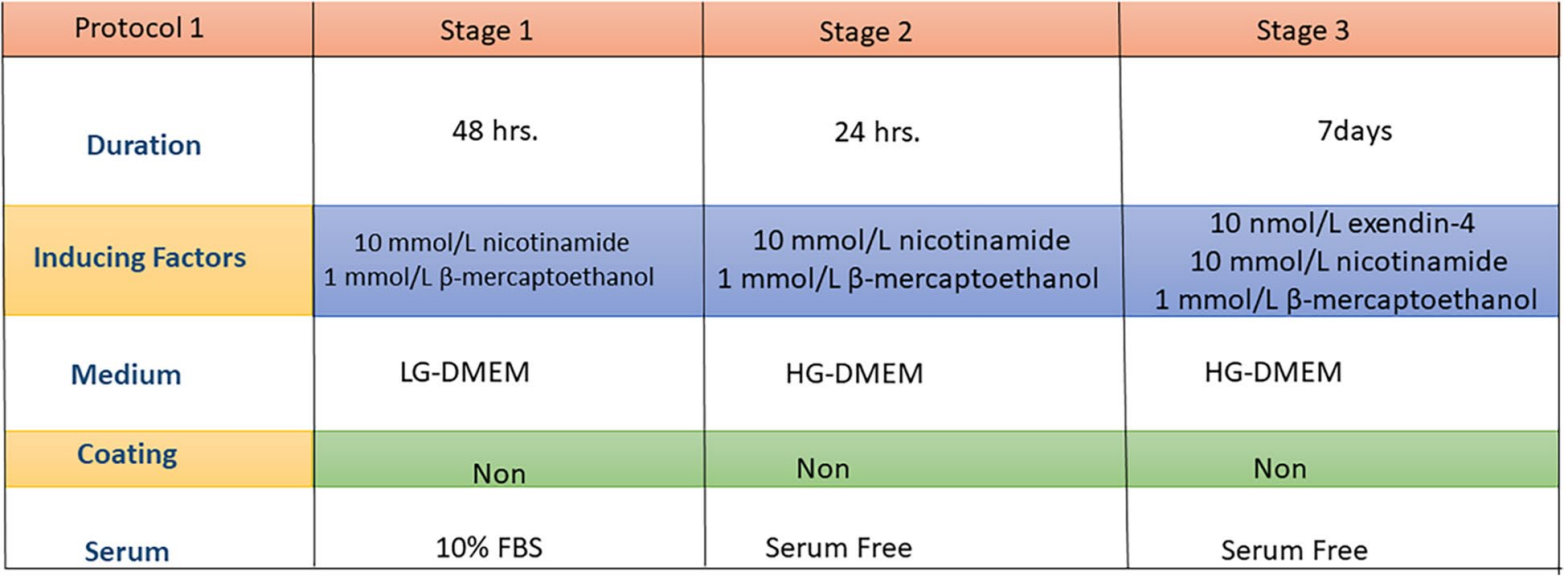
Schematic diagram outlining the differentiation protocol 1 for DPSCs & PDLSCs into pancreatic β cells. Factors required for each stage of differentiation are outlined

The first protocol was carried out as described by Kassem et al. in 2016 [16]. Briefly, 2 × 10^5^ dental derived stem cells were pre-induced for 48 hours with 10 mmol/L nicotinamide (Sigma-Aldrich, USA) and 1 mmol/L β-mercaptoethanol (Sigma-Aldrich) in 10% FBS LG-DMEM, and then re-induced for another 24 hours with 10 mmol/L nicotinamide and 1 mmol/L β-mercaptoethanol in serum-free high glucose (HG)-DMEM (Serox). This was followed by further induction for 7 days by 10 nmol/L exendin-4 (Sigma-Aldrich) in serum free HG-DMEM supplemented with 10 mmol/L nicotinamide and 1 mmol/L β-mercaptoethanol (Fig. 1).

#### Protocol 2

**Fig. 2:**
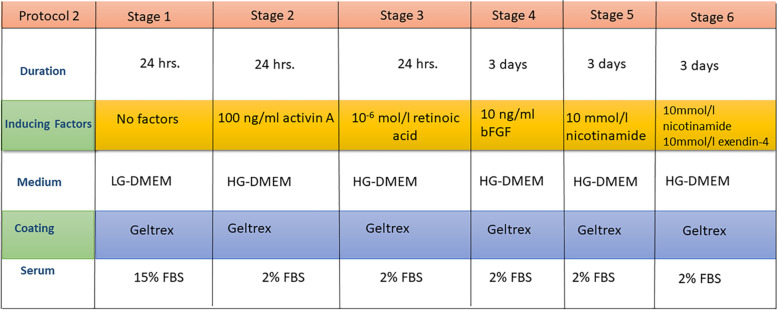
Schematic diagram outlining the differentiation protocol 2 for DPSCs & PDLSCs into pancreatic β cells. Factors required for each stage of differentiation are outlined

The second protocol was performed as designated by Wang et al. in 2012 [17]. First, 2 × 10^5^ dental derived stem cells were passaged on Geltrex™ (Gibco, USA)-coated 6-well culture plates in LG-DMEM containing 15% FBS for 24 h. Next, the culture medium was changed to HG-DMEM, containing 2% FBS and 100 ng/ml activin A (STEMCELL Technologies Inc., Canada) for 24 h, and then changed to HG-DMEM containing 2% FBS and 10^− 6^ mol/l retinoic acid (RA; Sigma Aldrich) for an additional 24 h. Thereafter, the cells were induced with HG-DMEM containing 2% FBS and 10 ng/ml basic fibroblast growth factor (bFGF; STEMCELL Technologies Inc). After 3 days of bFGF induction, the culture medium was changed to HG-DMEM containing 2% FBS and 10 mmol/l nicotinamide for 3 days. Finally, the cells were induced with HG-DMEM containing 2% FBS and 10 mmol/l nicotinamide in the presence of 10 mmol/l exendin-4 for 3 days (Fig. 2).

### Determination of successful differentiation into pancreatic β cell lineage

#### Real time PCR analysis of pancreatic β cells generated from differentiation of human DPSCs and PDLSCs

Total RNA was isolated from the pancreatic β cells generated from human DPSCs and PDLSCs using Trizol (Invitrogen) according to the manufacturer’s instructions. The first strand cDNA was synthesized with 50 ng of total RNA by random hexamer priming using high capacity cDNA synthesis kit (Qiagen) at 42 °C for 60 min and at 70 °C for 5 min. Quantitative real-time PCR was performed was carried out in triplicates with a SYBR-green kit (Qiagen) according to the manufacturer’s instructions and using the Roche LC480 in a total reaction volume of 20 μl. Results were normalized to GAPDH (internal control) to correct RNA input in reactions. All reactions were performed by annealing at 58–60 °C for 40 cycles and the melt curve analysis was achieved at the end of each reaction. Gene- specific primers are listed in Table 1.

**Table 1:** List of gene-specific primers in RT-PCR

Gene	Forward	Reverse
**Foxa-2**	GGAGCGGTGAAGATGGAAGG	CGGCGTTCATGTTGCTCAC
**Sox-17**	CAAGATGCTGGGCAAGTC	TGGTCCTGCATGTGCTG
**PDX-1**	ATGGATGAAGTCTACCAAAGC	CGTGAGATGTACTTGTTGAATAG
**Ngn-3**	AGAGAGCGTGACAGAGGC	GCGTCATCCTTTCTACCG
**INS**	GCTTCTTCTACACACCCAAG	GGTAGAGGGAGCAGATGC
**Gcg**	ACCAGAAGACAGCAGAAATG	GAATGTGCCCTGTGAATG
**GAPDH**	GCACCGTCAAGGCTGAGAAC	TGGTGAAGACGCCAGTGGA

#### Insulin release assay

Generated IPCs (2× 10^5)^ were gently washed twice with phosphate buffer saline (PBS, Biowest, France). Then, the cells were pre-incubated in culture media containing glucose (5.5 mM) at 37 °C for 24 h [19] and supernatants were then collected for insulin quantification via ELISA technique using the kit procured from Epitope Diagnostics, Inc. (USA) according to the manufacturer’s instructions. Results from three independent experiments were reported.

### Statistical analysis

The attained results are delineated as means ± standard deviations. Data were analyzed by one way analysis of variance (ANOVA) using the Statistical Package for the Social Sciences (SPSS) program, version 14 followed by least significant difference (LSD) to compare significance between groups. The level of significance was set at *P* < 0.05.

## Results

### Successful isolation of DPSCs & PDLSCs

**Fig. 3:**
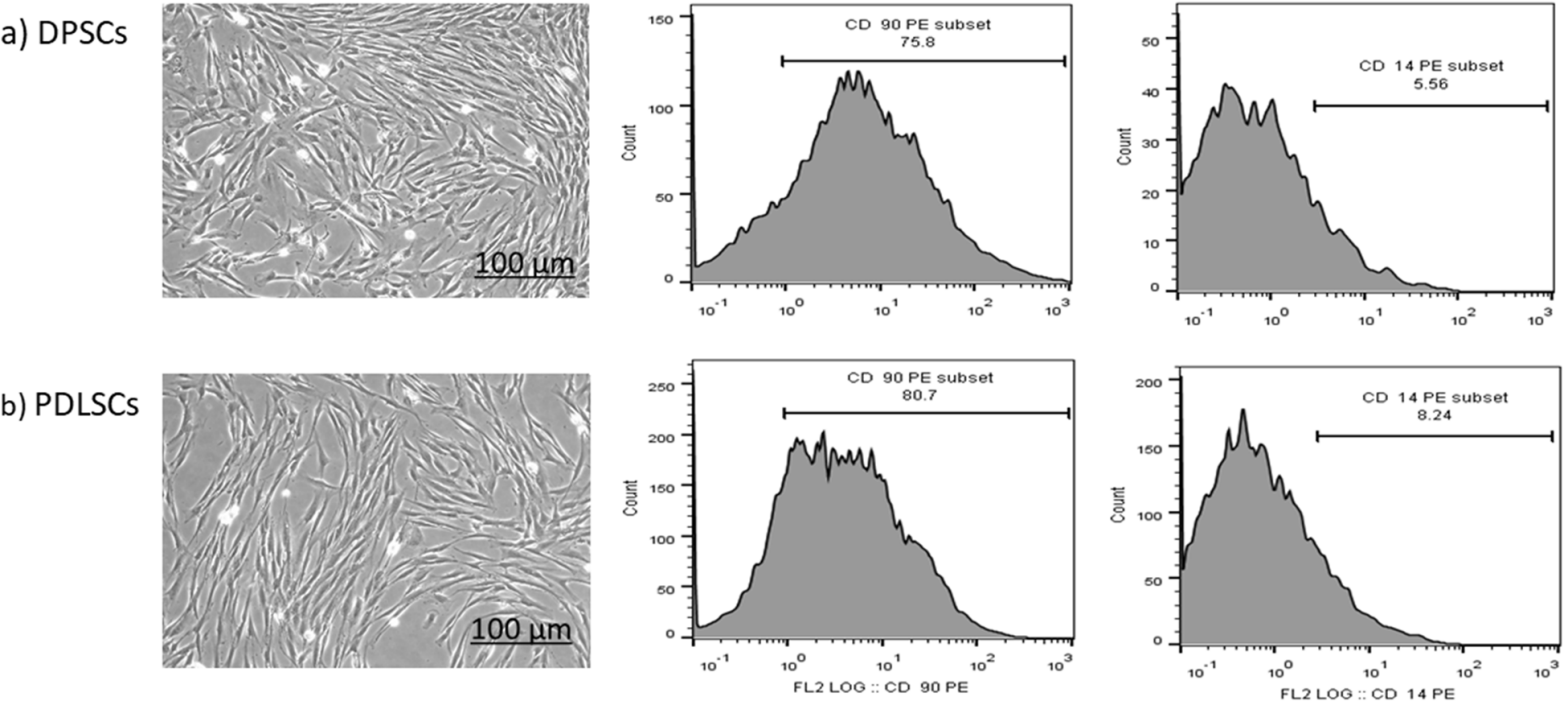
Successful isolation of DPSCs & PDLSCs. Typical morphological appearance of MSCs was illustrated in DPSCs (**a**) and PDLSCs (**b**). Cells assumed spindle shaped and stellate appearance with central whirlpool appearance as the cells increase in number. (Scale Bar 100 μm). Also, MSCs surface markers; CD 90 was positively expressed in both DPSCs (**a**) and PDLSCs (**b**) while CD 14 was negatively expressed in both DPSCs & PDLSCs

Morphological as well as phenotypic analysis confirmed the successful isolation of DPSCs & PDLSCs. After isolation, the cells assumed a typical fibroblastic, and spindle shaped appearance distinctive of MSCs with typical whirlpool appearance at confluence. Also, cells illustrated typical attachment to plastic culture plates. Moreover, the MSCs identity of both DPSCs & PDLSCs was confirmed by flow cytometric analysis. Both DPSCs and PDLSCs expressed MSCs marker CD 90 (75% & 80.7% respectively) and were negative for non-mesenchymal stem cell marker CD14 (5.56% & 8.24% respectively) (Fig. 3). Thus fulfilling the minimal criteria for defining multipotent mesenchymal stem cells [20, 21].

### Gene expressions of pancreatic β cells generated through differentiation of DPSCs & PDLSCs according to the implemented protocol of differentiation

**Fig. 4:**
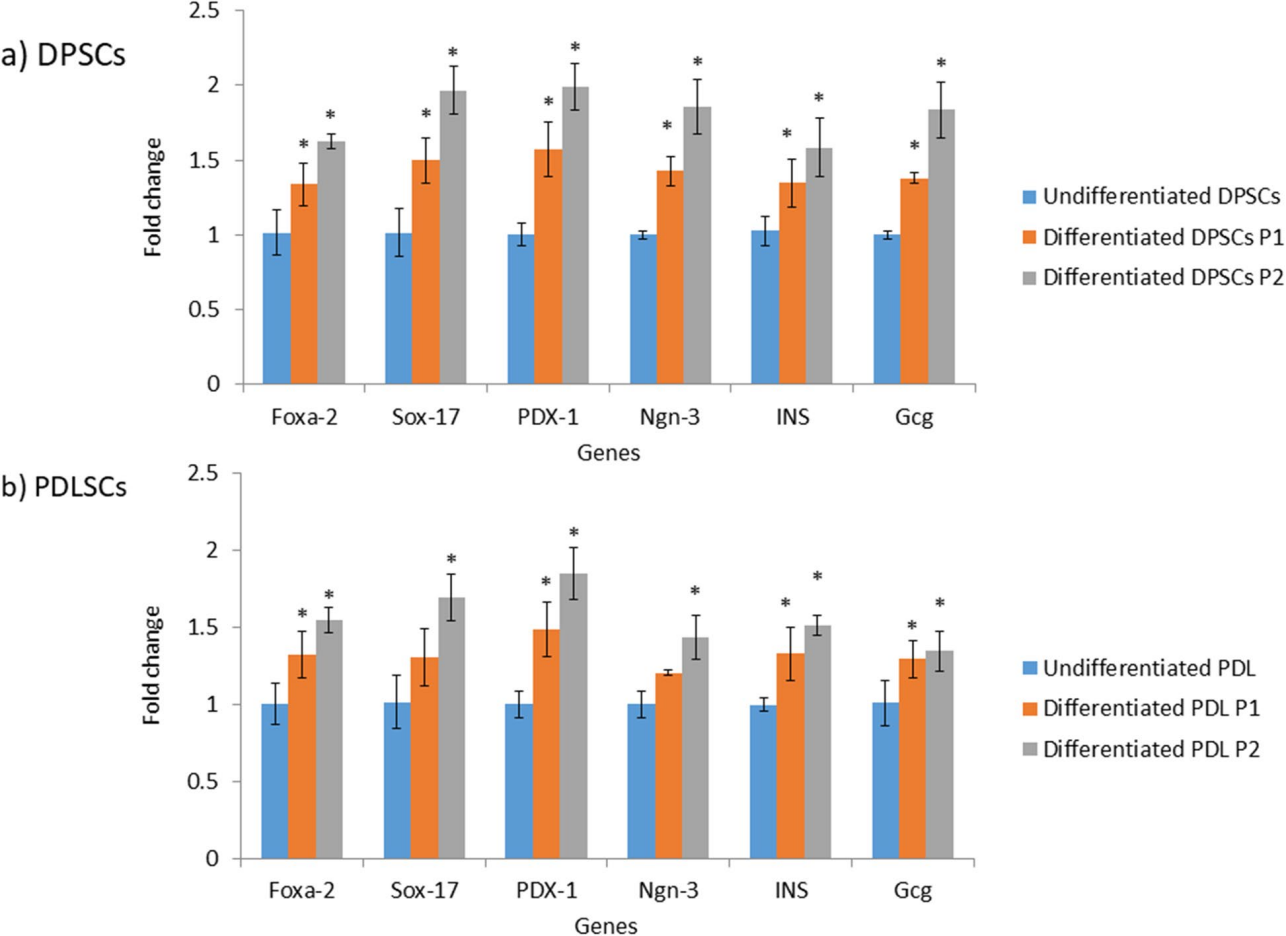
Expression of pancreatic endocrine genes in generated pancreatic β cells from DPSCs (**a**) and PDLSCs (**b**) by protocols 1 and 2. Data are expressed as Means ± SD. *Significant change at *P* < 0.05 in comparison with the undifferentiated counterparts

Figure 4a illustrated that human DPSCs that were induced to differentiate into IPCs through protocol 1 for 10 days or protocol 2 for 12 days showed significant (*P* < 0.05) up-regulation in the expression levels of *Foxa-2, Sox-17, PDX-1, Ngn-3, INS* and *Gcg* genes when compared with undifferentiated DPSCs. As for PDLSCs, by applying protocol 1 for 10 days on human PDLSCs, the generated cells demonstrated significant (*P* < 0.05) up-regulation in the expression level of *Foxa-2, PDX-1, INS* and *Gcg* genes versus the undifferentiated PDLSCs. On the other hand, the generated cells upon treatment of human PDLSCs by the inductive media described in protocol 2 for 12 days exhibited significant (*P* < 0.05) up-regulation in the expression levels of *Foxa-2, Sox-17, PDX-1, Ngn-3, INS* and *Gcg* genes in comparison with undifferentiated counterparts (Fig. 4b).

### Insulin release in response to glucose stimulation: in vitro

**Fig. 5:**
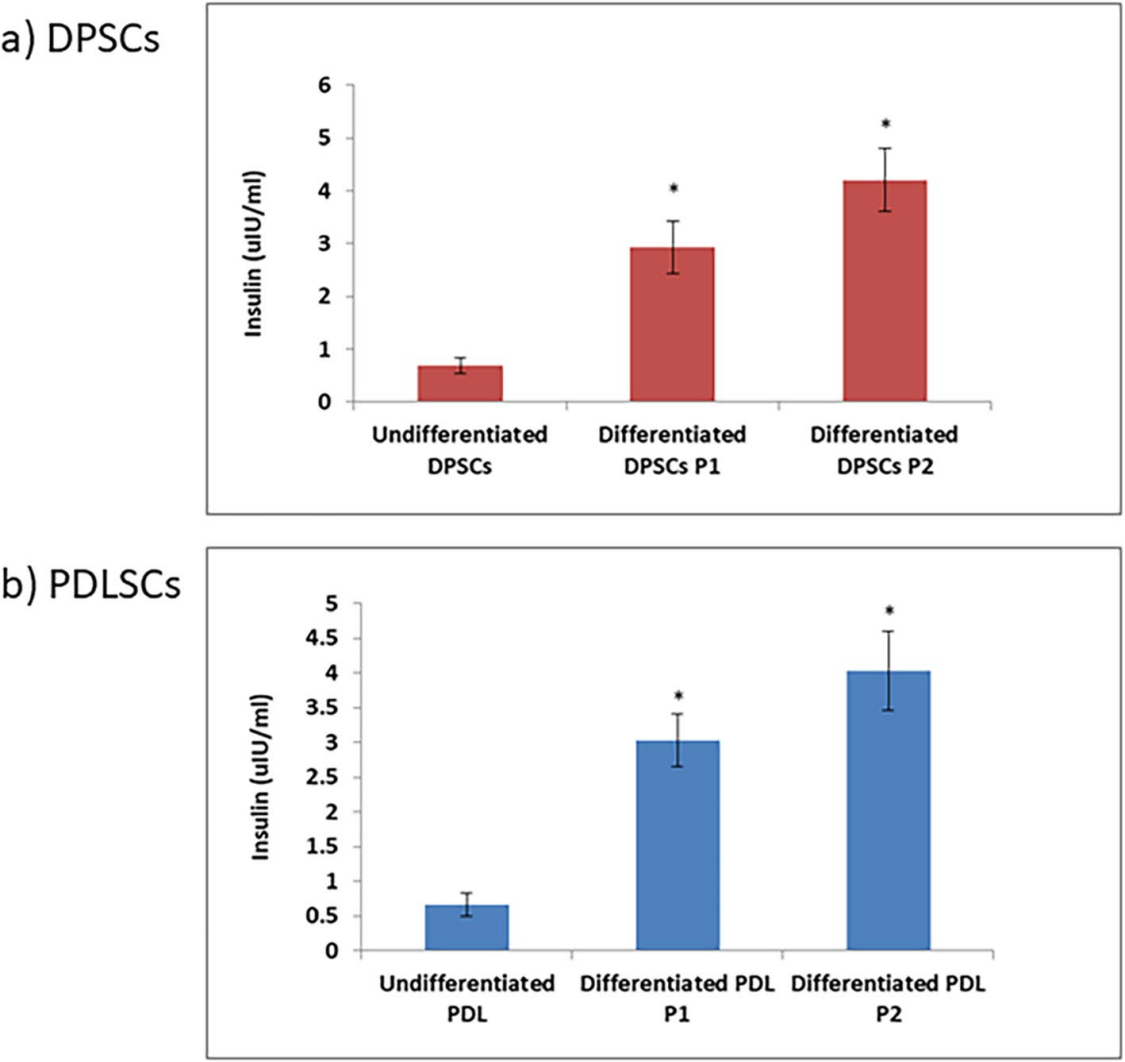
Insulin release of generated pancreatic β cells from DPSCs and PDLSCs by both protocols. Data are expressed as Means ± SD. *Significant change at *P* < 0.05 in comparison with the undifferentiated cells

The data in (Fig. 5) revealed that, the generated cells from differentiation of human DPSCs and PDLSCs by protocols 1 and 2 released significant (*P* < 0.05) amounts of insulin in response to increasing glucose concentration versus their undifferentiated counterparts.

### Comparison of pancreatic β cells differentiation capacity of DPSCS and PDLSCs

**Fig. 6:**
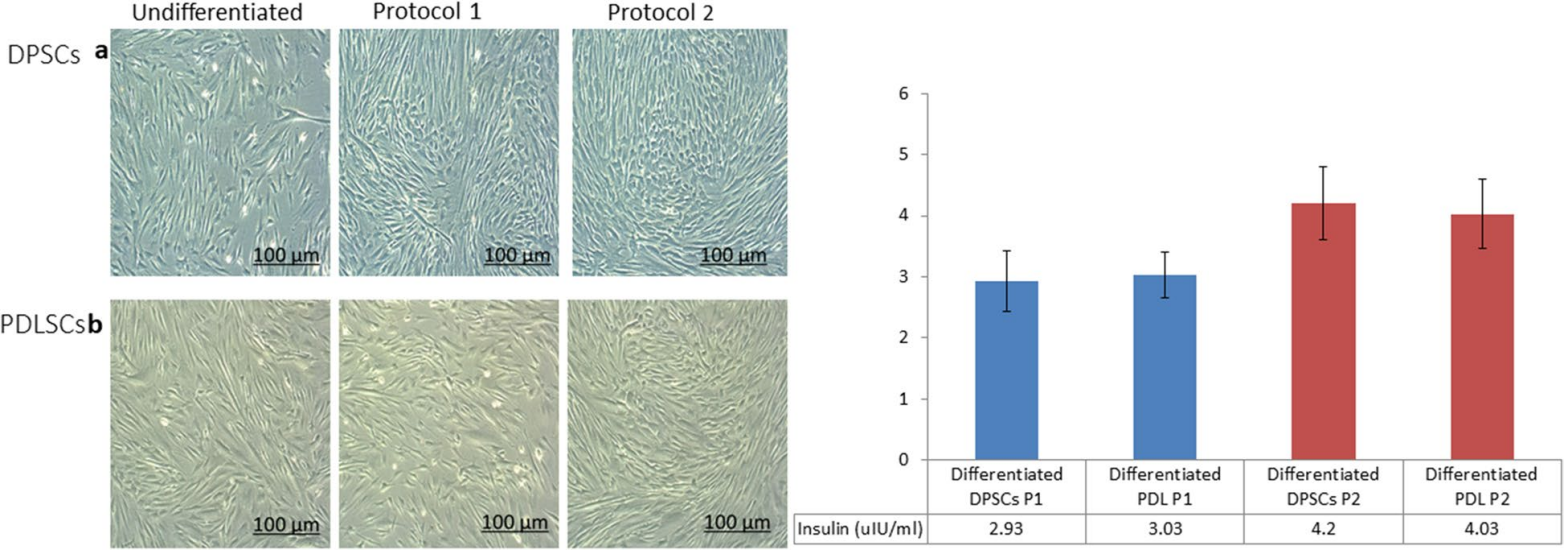
Morphological appearance & Insulin release assay of generated pancreatic β cells from DPSCs (**a**) and PDLSCs (**b**) after induction by both protocols. Following differentiation, both DPSCs (**a**) and PDLSCs lost their typical spindle shaped fibroblastic appearance and started to assume a more polygonal shape. No significant morphological difference between both cell types is apparent (Scale Bar 100 μm) (Left). Insulin release of generated pancreatic β cells from DPSCs and PDLSCs by both protocols (Right). Data are expressed as Means ± SD

The differentiation potential of DPSCs & PDLSCs under the two protocols applied was compared in order to evaluate the impact of the differentiation protocol on the type of cells used. At the end of the induction period, slight morphological changes from the classic spindle shaped fibroblastic appearance to more polygonal shape was observed in DPSCs & PDLSCs under both of the implemented differentiation protocols (Fig. 6).

**Fig. 7:**
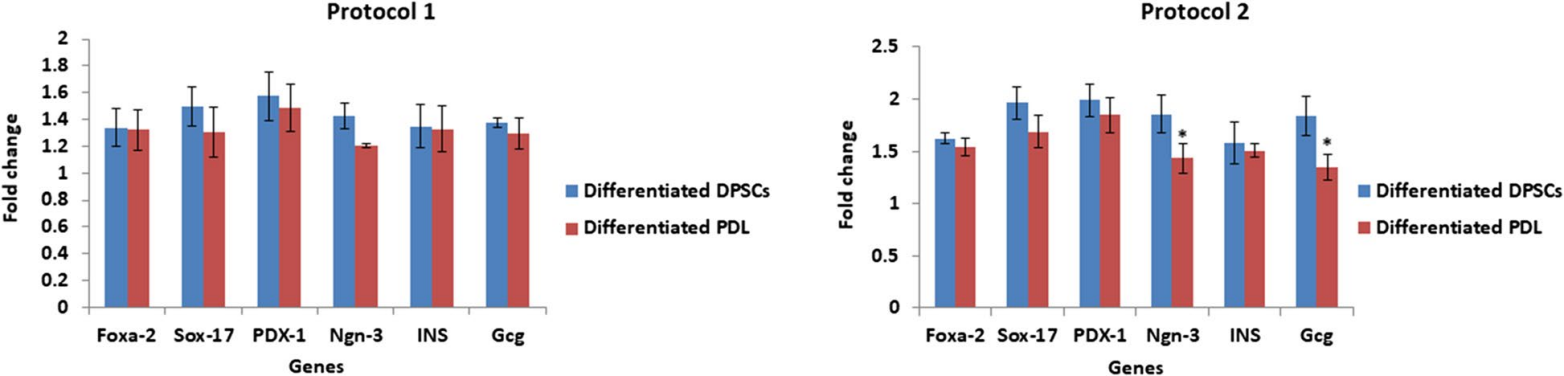
Comparison of pancreatic β cell differentiation capacity of DPSCS and PDLSCs by protocol 1 (**a**) and protocol 2 (**b**) by qRT- PCR. Data are expressed as Means ± SD. *Significant change at *P* < 0.05

As for the expression of pancreatic lineage markers by RT-qPCR, we compared the results of gene expression derived from both DPSCs & PDLSCs statistically in (Fig. 7). It was noticed that DPSCs illustrated increased levels of *Foxa-2, Sox-17, PDX-1, Ngn-3, INS* and *Gcg* genes expression when compared to PDLSCs differentiated under protocol 1. However, with no statistical significance. The same pattern was also expressed in protocol 2 where DPSCs illustrated higher expression of all the tested genes in comparison to PDLSCs. Significant downregulation of *Ngn − 3* and *Gcg* genes of PDLSCs versus DPSCs was observed (*P* < 0.05) (Fig. 7). The analysis of insulin release in response to glucose stimulation demonstrated that both DPSCs & PDLSCs possess the ability to differentiate into insulin producing pancreatic β cells. However, PDLSCs showed higher insulin release upon glucose challenge when compared to DPSCs under protocol 1 whereas DPSCs produced higher insulin levels contrary to PDLSCs using protocol 2 (Fig. 6). Overall, these results indicated that DPSCs exhibited an advanced potency for differentiation towards insulin producing pancreatic β cells than PDLSCs under either protocols.

## Discussion

Despite the tremendous efforts and recent progress in the differentiation strategies of stem cells into pancreatic beta cell lineage, current protocols are not optimized for different cell types. There is a need for establishment of efficient and reproducible protocols that are tailored for each and every cell source based on their regulatory intracellular pathways [22]. Further analysis of the differentiation potential of various stem cell sources into pancreatic beta cell lineage is thus indeed crucial along with evaluating the impact of various inducing agents on the differentiation process.

In the present research, we isolated stem cells from dental pulp and periodontal ligament and analysed their differentiation potential into pancreatic beta cell through applying two different established protocols designed for other MSCs.

Dental derived stem cells are consensually considered an excellent source of stem cells. Dental derived stem cells possess exceptional qualities that render them a promising therapeutic candidate in the field of regenerative medicine [23]. Both DPSCs & PDLSCs are autologously available cells that share a neural crest origin and express neuro ectodermal properties in addition to their multi-lineage differentiation capacity. Moreover, acquiring dental stem cells is a rather simple and straightforward procedure and presents no ethical controversy [24]. Another exceptional feature of dental derived stem cells is their favourable immuno-modulatory and anti-inflammatory properties which render these cells advantageous for allogenic transplantation in the clinical settings. A further aspect of dental derived stem cells is their ability to be cryopreserved for years while maintaining their proliferative and stemness characteristics [25].

Previous studies have concluded that dental derived stem cells are superior to other alternative sources of mesenchymal stem cells in generating pancreatic β cells since they can be easily obtained and present no ethical controversy especially if autologous transplantation is intended [8, 26–28]. Moreover, it has been reported that DPSCs can be differentiated into mature insulin producing cells capable of secreting insulin on stimulation, by contrast to bone marrow mesenchymal stem cells that were able to differentiate into IPCs, but were not able to secrete insulin [11]. Our results demonstrated that both DPSCs and PDLSCs exhibited mesenchymal stem cells characteristics and were successfully differentiated into pancreatic beta cell. Our findings regarding the ease of access and phenotype of the isolated DPSCs and PDLSCs were in accordance with the previous reports which further emphasize the valuable role of dental derived stem cells in numerous regenerative applications [29–33].

Considerable progress has been achieved in devising IPCs differentiation protocols suitable for potential clinical applications. In fact, a versatile range of differentiation protocols capable of generating IPCs from various sources of stem cells are now widely available [34]. However, the differentiation efficiency and the maturation capacity of those generated cells, are still faced with challenges that need to be addressed. With the purpose of improving the differentiation protocol for adipose derived mesenchymal stem cells, Dang le et al., proposed a three step differentiation protocol through manipulating the culturing microenvironment [35]. Also, in an attempt to mimic 3D cultures, Ojaghi et al., reported that the application of PLLA/PVA scaffolds significantly enhanced the differentiation potential of adipose derived mesenchymal stem cells into IPCs [36]. Similarly, Hosseinin et al., showed that silk/PES nano-fibrous scaffold aided in mimicking the in vivo microenvironment thus promoting the differentiation potency of mesenchymal stem cells into pancreatic beta cells lineage [37].

In the current approach, two protocols were investigated, the first protocol comprised culture conditions including exendin-4 along with increasing concentrations of nicotinamide and β-mercaptoethanol for a period of 10 days. On the other hand, the second protocol was supported by Geltrex basement membrane matrix for differentiation together with exendin-4, nicotinamide, bFGF, retinoic acid and activin A for a duration of 12 days. Both protocols successfully induced DPSCs and PDLSCs to differentiate into pancreatic beta cell lineage with no apparent differences morphologically, where both DPSCs & PDLSCs gradually lost their spindle shaped appearance and displayed a rather polygonal appearance. However, it was noted that in both DPSCs and PDLSCs, protocol 2 elicited stronger expression levels of all studied pancreatic markers (*Foxa-2, Sox-17, PDX-1, Ngn-3, INS and Gcg*) in comparison to undifferentiated DPSCs and PDLSCs. Furthermore, although we did not further investigate the generated cells in vivo*,* our in vitro data demonstrated the competence of the differentiated cells to secrete insulin in response to glucose challenge. Our results are in concomitant with Govindasamy et al., [11] who considered this finding an extremely important base for future clinical application. It is worthy to mention that both cells differentiated by protocol 2 illustrated higher response to glucose in comparison to those differentiated by protocol 1.

The outcomes of the present attempt clearly demonstrate the superiority of protocol 2 over protocol 1 in both cellular populations on the molecular level as well as on the functional level as evidenced by insulin secretion in response to glucose stimulation. It is important to point out that both protocols share the adminstration of exendin-4 as an inducting factor in at least one of the differentiation stages. Prior reports have indicated a favorable effect of exendin-4 on the differentiation potential of pluripotent stem cells and on enhancing the insulin release of IPCs [38, 39]. Additionally, it has been reported to promote MSCs proliferation and viability [40]. The mechanism of action of exendin-4 in inducing beta cell differentiation is not completely understood. It has been proposed that it activates *Pdx-1* through different intracellular pathways including phosphatidylinositol-3-kinase (PI3K), the hedgehog or the MAPK/ERK pathway [41]. Actually, this mechanism involving Pdx-1 induction was illustrated in our study as evident by the increased gene expression of Pdx-1 in both protocols. In fact, *Pdx-1* expression was the highest of all the pancreatic genes studied both in DPSCs and PDLSCs. It was reported that *Pdx-1* is an early marker of pancreatic development and beta cells synthesis [13, 42]. Nicotinamide was also a common inducing factor in both protocols. Its role in inducing islet generation from pancreatic progenitor cells and differentiation of liver stem cells into IPCs is well documented [43–48]. However, for initiation of protocol 2, DPSCs & PDLSCs were seeded on freshly coated Geltrex plates acting as an extracellular matrix to enhance stem cells differentiation into beta cells. Extracellular matrix plays a key role in directing stem cells towards differentiation especially in case of pancreatic cell lineage through presenting biochemical signals and growth factors [49]. Geltrex specifically is a soluble presentation of reduced growth factor basement membrane extract used for support of differentiation cultures. It is well known for its role in the differentiation of stem cells into pancreatic lineage [42] . Finally, retinoic acid was added in stage 3 of protocol 2 and acted as an activator of Pdx-1which was also reflected by upregulated Pdx-1 expression in both PDLSCs & DPSCs in Protocol 2.

Our results established that both DPSCs and PDLSCs exhibited mesenchymal stem cells characteristics and were successfully differentiated into pancreatic beta cell. Meanwhile, we analysed our results further in order to compare the differentiation potency of each type of cell (DPSCs & PDLSCs) under either protocols. From the present results, it was evident that DPSCs possessed higher tendency to differentiate towards pancreatic lineage than PDLSCs as illustrated by both the molecular analysis and the insulin release assy. These findings are in agreement with Shivakumar et al., who proposed that stem cells isolated from different anatomical parts of the dental tissues possessed variable degrees of differentiation potential [9]. Their study compared the potential of stem cells isolated from dental pulp, papilla, and follicle to differentiate into pancreatic lineage. The differentiation protocol implemented in that study was based on Geltrex as well, however, they did not include PDLSCs unlike the present study. Also, from the results obtained in the current study, it could be concluded that the selection of the protocol according to the cells being differentiated is crucial. It can be speculated that extrinsic factors like ECM can act synergistically with different cytokines and growth factors to enhance the process of differentiation. Accordingly, it is recommended to thoroughly study and apply the combinations of these extrinsic factors together in order to achieve efficient and functioning beta cells.

The present study can thus be considered a step forward towards considering optimized and well validated protocols for differentiation of pancreatic beta cells through careful analysis of the starting cell population and the applied protocol. However, it is worthy to mention that the results of this report exhibited some limitations that need to be addressed in future research. Although the present study demonstrated that both DPSCs & PDLSCs were able to differentiate into IPCs, differentiated cells secreted low levels of insulin upon stimulation in comparison to similar published data derived from healthy human pancreatic cells. This could be explained by the incomplete maturation of the derived β cells in vitro due to the lack of mesenchymal epithelial interaction that is normally present in vivo. Another aspect to be considered, is the purification of the final differentiated cell population. In our study, the molecular markers expression revealed a rather heterogeneous cell population. This should be addressed in future studies through optimizing the differentiation protocols in order to minimize the presence of cells in different maturation stages. Furthermore, we intend to carry out further future research utilizing our generated IPCs in an in-vivo experimental diabetic model in order to fully investigate both the glucose level secretion and the immunomodulatory influences of these cells.

In conclusion, the current report provides a clear scientific evidence for the promising potential of dental derived stem cells in differentiating into pancreatic beta cell lineage through selection of the right protocol, thus presenting a non-controversial and noninvasive cell source for beta cell generation that can be safely used for autologous cell based therapy for diabetes mellitus.

## Data Availability

The datasets generated during and/or analyzed during the current study are available from the corresponding author on reasonable request.
